# Microvascular invasion (MVI) is a poorer prognostic predictor for small hepatocellular carcinoma

**DOI:** 10.1186/1471-2407-14-38

**Published:** 2014-01-24

**Authors:** Min Du, Lingli Chen, Jing Zhao, Feng Tian, Haiying Zeng, Yunshan Tan, Huichuan Sun, Jian Zhou, Yuan Ji

**Affiliations:** 1 Department of Pathology, Zhongshan Hospital, Fudan University, Shanghai 200032, China; 2 Liver Cancer Institution, Zhongshan Hospital, Fudan University, Shanghai 200032, China

**Keywords:** Small hepatocellular carcinoma, Microvascular invasion, a-fetoprotein, Clinical features, Pathological features

## Abstract

**Background:**

Small hepatocellular carcinoma (SHCC) is a special type of hepatocellular carcinoma with the maximum tumor diameter ≤ 3 cm and excellent long-term outcomes. However, the prognostic factors for SHCC remain controversial. The purpose of this study is to clarify the predictive factors of SHCC.

**Methods:**

The study population consisted of 458 patients underwent hepatectomy for single SHCC between January 2006 and December 2008. Clinical data (included age, gender, virus infection, serum alfa-fetoprotein level, cirrhosis, capsule, border), histopathologic features (included differentiation, morphology subtype, microvascular invasion, tumor infiltrative lymphocytes (TIL), inflammatory injury grade and fibrosis stage of surrounding liver), were evaluated to identify prognostic factors influencing SHCC patients’ survival and microvascular invasion.

**Results:**

There were 384 males (83.8%) and 74 (16.2%) females with median ages of 52 years. The median progression-free survival (PFS) and overall survival (OS) durations were 53 and 54 months, respectively. About 91.9% (n = 421) SHCC were infected by Hepatitis B. One hundred forty-seven of the 446 (33.0%) patients with pre-operation serum AFP level record had serum alfa-fetoprotein (AFP) level ≥ 200 ug/ml and 178 of the 280 (63.8%) patients with post-operation serum AFP level record had AFP level ≥ 20 ug/ml. Liver cirrhosis was present in 411 cases (89.7%), while 434 (97.3%) tumors had clear border, and 250 (55.6%) tumors were encapsulated.

MVI was identified in 83 patients (18.1%). In univariate analysis, a significant association between the presence of MVI and shortened PFS and OS was found (p = 0.012, 0.028, respectively). Histological differentiation had strong relationship with MVI (p = 0.009), in terms of MVI was more easily presented in patients with worse histological differentiation. In patients with MVI, worse survival was correlated with female patients, patients with G2 or G3 histological differentiation, pre-operation serum AFP level ≥ 200 ug/ml or post-operation serum AFP level ≥ 20 ug/ml, and TIL ≥ 50/HPF.

**Conclusions:**

MVI is an independent poorer prognostic factor for PFS and OS of single SHCC patients. Tumor histological differentiation was closely related with MVI.

## Background

Hepatocellular carcinoma (HCC) is the fifth most common malignancy and the third cause of cancer-associated death worldwide, with the increase of incidence and mortality every year [1]. Patients with solitary HCC up to 3cm has been reported to be less aggressive and characterized by excellent long-term outcomes after surgical resection in several studies. The size cutoff of 3 cm has been first adopted to define SHCC in the Pathological Classification of Liver Cancer in 1979 and the latest edition of the Consensus of Diagnosis and Treatment of Primary Liver Cancer in 2009 in China followed the definition [2]. In addition to tumor size, worse histological differentiation, higher tumor stage, and presence of any of the following: microvascular invasion (MVI), intrahepatic metastasis, tumor rupture or portal venous invasion were significant risk factors for immediate post-operative recurrence of HCC [3]. Despite remarkable improvement in surgical techniques and perioperative management, the long-term outcome after resection of SHCC is far from satisfactory because of the higher post-operative recurrence.

The assessment of the impact factors for small hepatocellular carcinoma represents a hot-topic issue that requires further investigation and clarifications. The present study was performed to identify the risk factors for recurrence and survival of SHCCs.

## Methods

This study was conducted in accordance with a protocol approved by the institutional review board of Zhongshan Hospital, Fudan University.

All SHCC patients (1376) were confirmed from routing diagnostic criteria from 3467 patients treated with liver resection for liver space-occupying masses (1376/3467, 40%) in Liver Cancer Institution, Zhongshan Hospital between 2006 and 2008. Five hundred and thirteen patients with complete clinicopathological and follow-up data from 1376 patients were chosen for analysis. Fifty-five patients with multiple tumors were excluded from the study. In all, 458 SHCC patients were reviewed to investigate the prognostic factors of SHCC in our study.

The following clinicopathological and surgical variables were evaluated for their influence on progression-free (PFS) and overall survival (OS): age, gender, disease etiology, alfa-fetoprotein (AFP) level, tumor capsule, border, histological differentiation, morphology subtype, fatty change, tumor infiltrative lymphocytes (TIL) MVI, inflammatory injury grade and fibrosis stage of surrounding liver.

Serologic presence of any hepatitis B antigen or antibody was considered to be positive evidence of hepatitis B virus (HBV). The serologic presence of hepatitis C antibody was considered as evidence of positive for hepatitis C virus (HCV). Tumor size was based on the largest dimension of the tumor recorded by surgeon. Tumor grade was assessed using the scheme outlined by Edmondson and Steiner and was recorded based on the highest grade in a specimen [4]. Microvascular invasion (MVI) was defined as presence of tumor emboli in a portal radicle vein, large capsule vessel or in a vascular space lined by endothelial cells [5]. TIL were evaluated by counting the number of lymphocytes in tumor areas adjacent to surrounding liver microscopically. The degree of fibrosis was assessed on the basis of the Ishak score, and grades F5 and F6 were considered cirrhosis [4].

### Follow-up

Patients were followed up by the Liver Cancer institution, every three months by tumor marker analysis (AFP) and ultrasound or computed tomography at least every 6 months for more than two years. The last date of follow-up is July 6th, 2012. Mean follow-up was 54 months (4~75 months). Patients who had tumor recurrence were treated with re-resection when possible or by transcatheter arterial chemoembolization, percutaneous ethanol injection, radiofrequency ablation or radiotherapy. Progression free survival (PFS) was defined as the number of months from the date of surgery to the first documentation of disease recurrence or progression. Disease progression or recurrence status was determined on the basis of objective imaging according to RECIST criteria. Disease-specific overall survival (OS) was defined as the number of months from the date of surgery to the date of the last follow-up visit or time of death attributed to HCC.

### Statistical analysis

Statistical Analysis was performed using IBM SPSS software (version 16.0, SPSS Ink). We performed analysis of survival with Kaplan-Meier curves. Significant variables were tested in multivariate analysis using Cox proportional hazads regression model. Statistical significance was considered reached when p-value was below 0.05. Pearson X^2^ test was used for the assessment of variables associated with MVI. Peri-operation deaths were included in the analysis of overall survival results but excluded from the analysis of progression-free survival. Relative Risk (RR) and Odds Ratio (OR) were calculated using χ^2^ test.

## Results

### Clinicopathological characteristics

**Figure 1:**
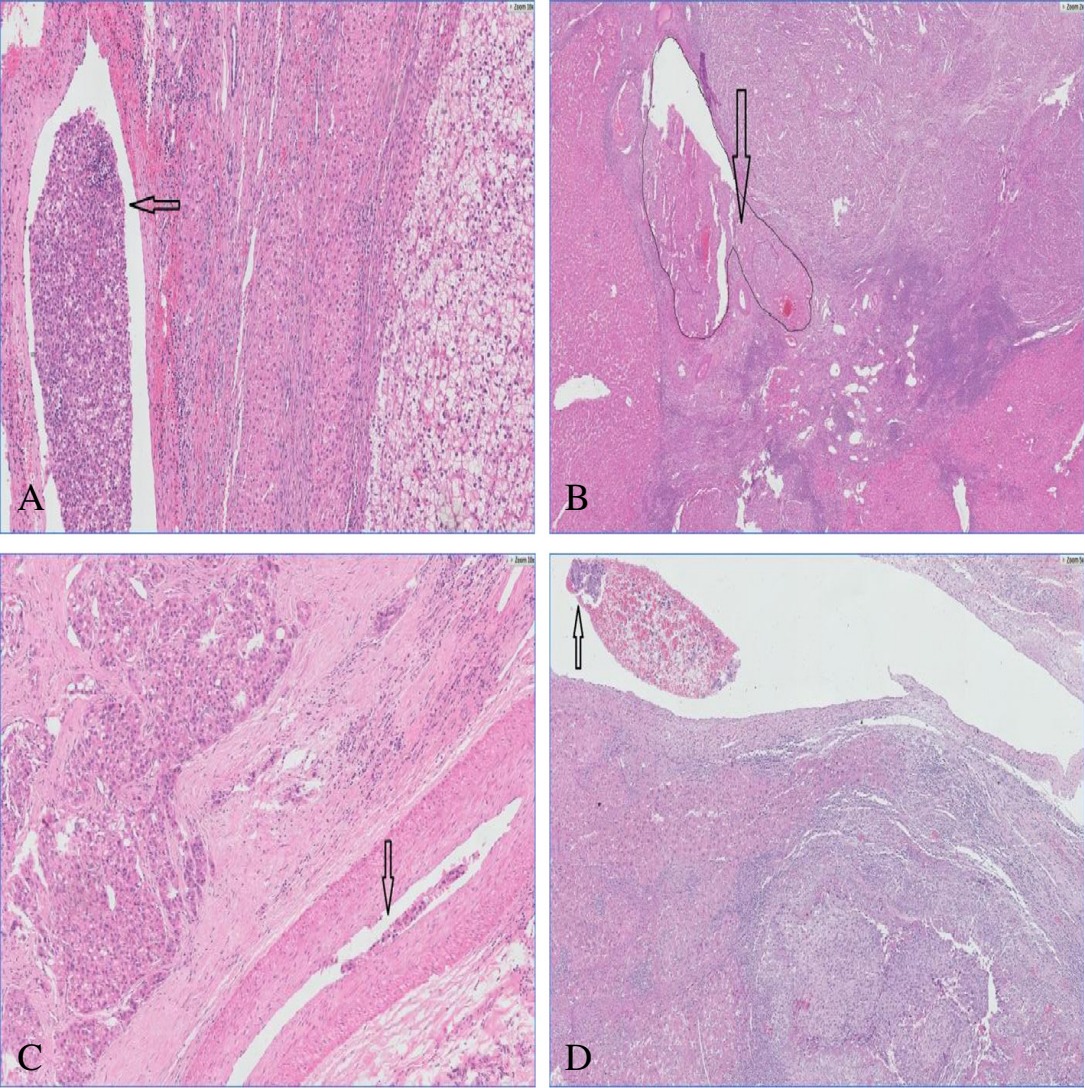
**Mrophological features of MVI.** Tumor thrombus were found in vessels of surrounding liver in **A** SHCC without capsule (HE ×100); **B** SHCC with infiltrative lymphocytes ≥ 50/HPF (HE ×200); **C** SHCC with invasive border and incomplete capsule (HE ×100); **D** poorly differentiated SHCC (HE ×50).

The clinicopathologic data of the patients cohort are summarized in Table 1. There were 384 males and 74 females with male to female ratio approximately 4.8:1. Mean age at diagnosis was 52.7 (10~87) years. HBV was the etiologic agent in 421 (91.9%) patients, four (0.9%) patients infected with HCV, one (0.2%) patient co-infected with hepatitis B and C, the other one (0.2%) patient co-infected with hepatitis B and E. Thirty (6.6%) patients without virus infection. Among the 446 patients with pre-operation serum AFP level record, 147 (33.0%) patients had serum AFP level ≥ 200 ug/ml. Of the 280 patients with post-operation serum AFP level record, 178 (63.6%) patients exhibited post-operation serum AFP level ≥ 20 ug/ml. Of the 449 patients with capsule record, 249 (55.5%) tumors were encapsulated, 200 (44.5%) tumors with partial capsule. A total of 434 tumors had well-demarcated border. Liver cirrhosis was presented in 411 (89.7%) cases. Nine (2.0%) cases were observed with schistoma eggs in the portal areas. MVI was identified in 83 (18.1%) patients. MVI was observed at a magnification of 200× with cluster tumor cells gathering in a vascular space with/without smooth muscle wall, and mixed with blood cells (Figure 1).

**Table 1:** Clinicopathological characteristics of 458 patients with single SHCC

**Clinicopathological features**	**Value**^**a**^
Age(yr), mean (median)	52.65 (52)
Sex	
Male	384 (84.0%)
Female	74 (16.0%)
Pre-operation AFP value ≥200 ug/ml^a^	147 (33.0%)
Post-operation AFP value ≥20 ug/ml^b^	178 (63.6%)
Virus infection^c^	
No virus	30 (6.6%)
HBV	421 (92.0%)
HCV	4 (1.0%)
HBV&HCV	1 (0.2%)
HBV&HEV	1 (0.2%)
Cirrhosis	
Y	411 (89.7%)
N	47 (10.3%)
Capsulary formation^d^	
Y	249 (55.5%)
N	200 (44.5%)
Tumor border^e^	
Clearly	434 (97.3%)
Vaguely	12 (2.7%)
Tumor histological differentiation	
Well differentiated	14 (3.1%)
Moderately differentiated	345 (75.3%)
Poorly differentiated	99 (21.6%)
Tumor microscopic manifestation	
MVI	
Y	83 (18.1%)
N	375 (81.9%)
Tumor infiltrative lymphocytes	
≥50/HPF	84 (18.3%)
<50/HPF	374 (81.7%)
Clear cell subtype	
Y	23 (5.0%)
N	435 (95.0%)
Tumor fatty change	
Y	15 (3.3%)
N	443 (96.7%)
Large cell dyplastic change	
Y	36 (7.9%)
N	422 (92.1%)
Peritumor liver fatty change	
Y	90 (19.7%)
N	368 (80.3%)
PFS(mo),median(range)	53.00 (4-75)
OS(mo),median(range)	54.00 (21-75)

^a^Missing data in 12 cases.

^b^Missing data in 178 cases.

^c^Missing data in 1 cases.

^d^Missing data in 9 cases.

^e^Missing data in 12 cases.

**AFP** a-fetoprotein, **MVI** microvascular invasion.

^a^Values are expressed as n(%) or median(range).

### Impact factors of long-term survival

Among 458 patients, 102 (22.3%) patients experienced HCC recurrence or de novo tumors. Eighty-four (18.3%) patients died within 5 years follow-up, among which 50 (59.5%) patients had HCC recurrence. The median PFS and OS durations were 53 and 54 months, respectively. The 5- year PFS and OS rate was 77.7% and 81.7%, respectively.

**Figure 2:**
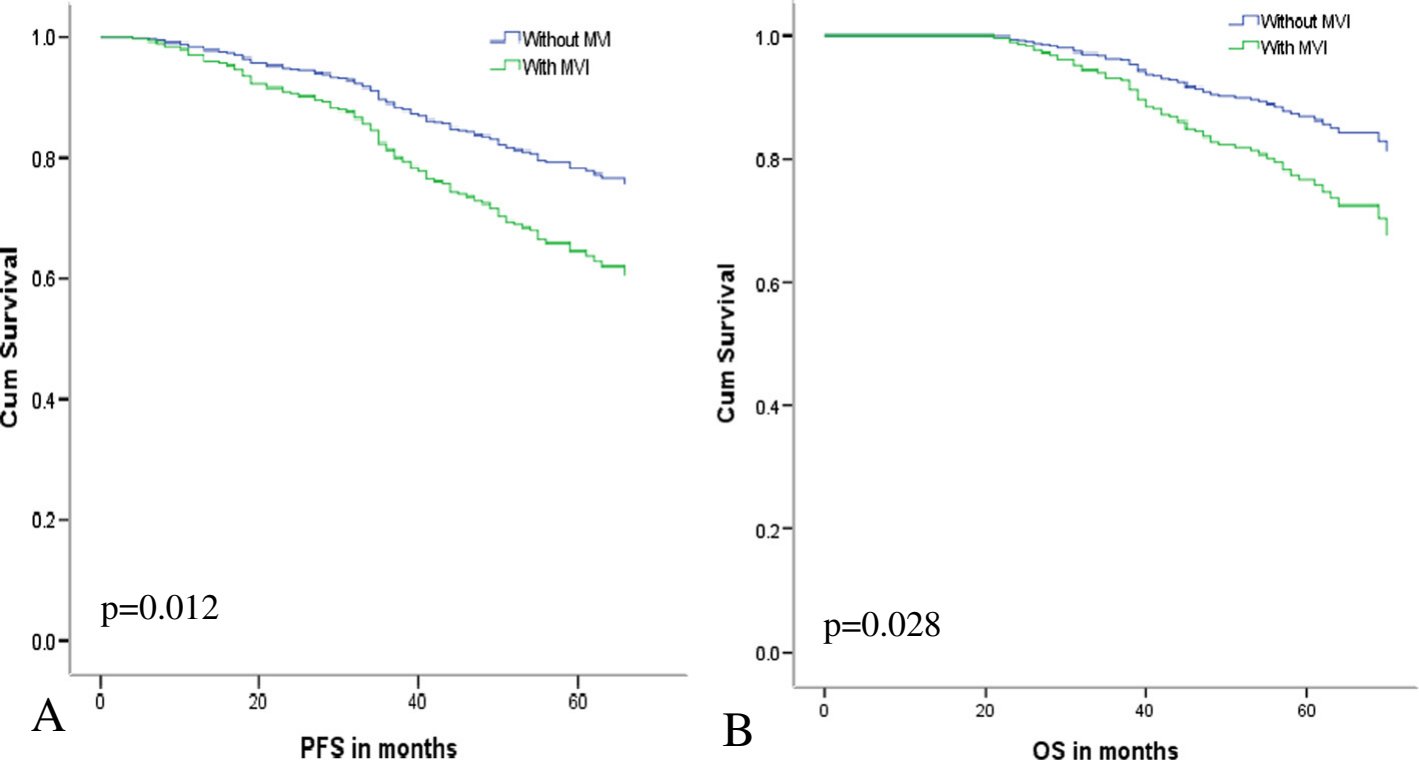
**Cox Regression analysis of SHCC patients with MVI.** 5 year cumulative survival of patients with MVI after hepatectomy, **A** PFS (p = 0.012); **B** OS (p = 0.028).

Univariate analysis suggested that SHCC patients with MVI had shortened PFS and OS (p = 0.012, 0.028, respectively) (Figure 2). Kaplan-Meier survival analyses revealed that MVI was associated with poorer PFS and OS for females (p < 0.001, PFS&OS, both), patients with G2 or G3 histological differentiation (p = 0.017, 0.008, PFS&OS, respectively), pre-operation serum AFP level ≥ 200ug/ml (p = 0.022, 0.014, PFS& OS, respectively), or post-operation serum AFP level ≥ 20 ug/ml (p = 0.014, 0.003, PFS&OS, respectively), as well as patients with TIL ≥ 50/HPF in tumor areas (p = 0.001, p = 0.006, PFS&OS, respectively) (Table 2).

**Table 2:** Impact of MVI and following factors for SHCC patients’ survival

**Variables**	**With MVI**	**Without MVI**	**p value**^**1**^**(log-rank)**	**p value**^**2**^**(log-rank)**
Sex				
Male	69	315	0.257	0.223
Female	14	60	0.002	0.000
Age(yr)				
≥50	44	218	0.311	0.081
<50	39	157	0.028	0.095
Pre-operation AFP value (ug/ml)				
≥200	25	122	0.022	0.014
<200	55	244	0.370	0.330
Post-operation AFP value (ug/ml)				
≥20	34	144	0.021	0.003
<20	19	82	0.339	0.633
Cirrhosis				
Y	75	336	0.023	0.056
N	8	39	0.747	0.174
Capsule				
Y	39	211	0.798	0.810
N	41	159	0.008	0.006
Tumor border				
Y	78	356	0.012	0.035
N	4	8	0.051	0.091
Tumor infiltrative lymphocytes(/HPF)				
≥50	10	74	0.001	0.006
<50	73	301	0.065	0.114
Tumor differentiation				
G1	2	12	0.752	0.752
G2&G3	81	363	0.008	0.017

p value^1^: p value of factors affecting patients’ PFS.

p value^2^: p value of factors affecting patients’ OS.

**AFP** a-fetoprotein, **MVI** microvascular invasion.

### Impact factors of microvascular invasion

Pearson X^2^ analysis indicated that histological differentiation (p = 0.009) was closely related with MVI. In well or moderately differentiated tumors, 15.6% with MVI, compared to 27.0% in poorly differentiated tumors. Increasing tumor size was also associated with higher rates of MVI.

## Discussion

Evidence had suggested that tumor size is one of the most important prognostic factors of patients with hepatocellular carcinoma (HCC) [6]. The latest Consensus on Diagnosis and Treatment of Primary Liver Cancer in China adopted 3 cm as the definition of small HCC [2]. Increasing research showed that when HCC is about 3 cm in size may be important as changes occur in DNA stemline and biological characteristics, and HCC > 3 cm exhibited a tendency towards more aggressive behavior and may reach an important turning point for critical transformation with a resultant change to a more infiltrative behavior [7,8].

It is generally accepted that the incidence of HCC is higher in men than in women. Gender was not an independent prognostic factor for SHCC patients in our study, however, MVI displayed survival differences in female patients other than in males. The comparison of variables showed that AFP had a positive correlation with gender. Differences in several aspects of medical management may contribute to the gender disparity in survival rates. Underlying mechanism is still obscure and optimal therapeutic regimens or hormonal mechanisms regarding HCC development should be elucidated to improve clinical outcomes [9].

AFP has served as a representative tumor marker of HCC for more than 40 years. Liu et al. confirmed that AFP levels were remarkably higher in patients with vascular invasion (P < 0.001) [10]. High pre-operation and post-operation AFP level has certain relationship with long-term survival and MVI. While AFP level is positively associated with tumor size, the larger tumor the higher AFP, it is reasonable that AFP level failed to show a prognostic value for survival of SHCC patients.

Microvascular invasion is a histological feature of hepatocellular carcinoma related to aggressive biological behavior. HCC is characterized by a tendency for vascular invasion. During the past decades, many studies have addressed the prognostic significance of MVI in HCC, either as a primary or secondary object. Nevertheless, the prognostic significance of MVI remains controversial, and there is a significant interobserver and intraobserver variability in the assessment of MVI. Junichi et al claimed that microvascular invasion does not affect survival of SHCC (up to 2 cm) [11], others with opposite opinion that microscopic vascular invasion was an independent factor for SHCC (up to 3 cm) [12]. Our findings indicate that MVI has an adverse impact on long-term survival in SHCC patients. Presence of MVI led to a significant decrease in PFS and OS at 5 years. Combined our results with other four studies included in Manuel Rodrıguez- Peralvarez et al meta-analysis (n = 1959), the RR of MVI to PFS survival was 1.34 (95% CI =1.24-1.51) [5]. An international consensus delineating what is meant by MVI in HCC could provide a more consistent evaluation and, therefore, a more reliable prediction of prognosis and a better understanding of the pathophysiology of HCC angioinvasion.

Because microvascular invasion is a histopathologic diagnosis, it cannot be made prior to the resection of the tumor. Given the fact that MVI has a significant impact on recurrence and survival after hepatectomy, preoperative means of assessing the probability of MVI are needed. Increasing tumor size and AFP level were also recorded to be associated with higher rates of MVI [13,14] in others research. In current study, we found that tumor grade is a strong predictor of MVI. It’s well-proved that histological grade was an important prognostic factor for survival of HCC patients [15]. It has a strong relationship with MVI but has no statistically significance on survival, since most of the patients were moderately differentiated in our study. It indicated that SHCC may be not an early-stage of HCC, it certainly represents an earlier lesion. A significant correlation between infiltrative tumor margin in preoperative CT and MVI was claimed, indicating tumor margin may serve as a radiological sign in prediction of MVI in HCC patients [16].

Tumor gross features including cirrhosis, capsule and border failed to show prognostic impact on patients’ survival. Liver resection in the presence of compensated liver cirrhosis is feasible but associated with a significantly unfavored prognosis for overall and progression-free survival. Therefore preventing the progression of cirrhosis are important methods to improve the survival of HCC patients [17,18].

None of morphological features of surrounding liver correlated with MVI and patients’ survival, including peritumor large cell dysplastic foci (LCD), fatty change of hepatocytes. LCD was originally considered a precursor lesion of HCC and, accordingly, was referred to as dysplastic. Based on genetic and animal tests, Ferrell thought that LCD were pathogenetically linked to and associated with HCC but do not represent a direct precursor of HCC [19]. Tumor fatty change was more easily observed in patients with well differentiation (Grade I-II), a significant proportion of small (<2 cm) or “early” HCCs that are only vaguely nodular have been observed to have diffuse fatty change [20].

HCC is an example of inflammation-related cancer and represents a paradigm of the relation occurring between tumor microenvironment and tumor development. Stromal cells in tumor microenvironment secrete cytokines and proteins that promote angiogenesis, metastasis may contribute to MVI.

The prognosis of HCC patients remains unsatisfactory although it has been improved much in the past decades. For SHCC patients, resection is considered the most effective treatment. However, recurrence is the leading cause of death during the initial 5-year period after intensive radical resection. High possibility of intrahepatic recurrence remains one major obstacle for further improving the survival and prognosis of SHCC patients after curative resection. As MVI was a pivotal impact factor for SHCC, the detection and prevention for MVI will be a target for the therapy of SHCC patients. We will dedicated in developing the model for prediction of HCC patients’ survival and exploring measures to prevent recurrence or metastasis. For those patients with high risk factors of recurrence, intensive follow-up with serum AFP and radiology is one of the best methods to be recommended.

## Conclusions

In conclusion, the present study confirmed that microvascular invasion has adverse effect on single small hepatocellular carcinoma patients’ survival and more easily discovered in worse differentiated and large tumor patients, we wish to establish a model which can predict and improve the survival of HCC patients.

### Consent

Written informed consent was obtained from the patient for the publication of this report and any accompanying images.

## Competing interests

All the authors declare that they have no competing interests.

## Authors’ contributions

Conception and design: YJ. Acquisition of data: MD, HCS. Analysis and interpretation of data: MD, JZ. Draft the manuscript: MD. Statistical analysis: MD. Critical revision of the manuscript for important intellectual content: YJ. Technical or material support: LLC, HYZ, JZ, FT. Study supervision: YJ. All authors read and approved the final manuscript.

## Pre-publication history

The pre-publication history for this paper can be accessed here:

http://www.biomedcentral.com/1471-2407/14/38/prepub
